# Is there a difference in surgical accuracy following bimaxillary surgery between cleft and non-cleft patients?

**DOI:** 10.1007/s00784-024-05517-5

**Published:** 2024-01-24

**Authors:** Dirk-Melle Beek, Dirk-Jan Visser, Yi-Hsuan Chen, Frank Baan, Marloes Nienhuijs, Tong Xi

**Affiliations:** 1 Department of Oral and Maxillofacial Surgery, Radboud University Medical Center, Geert Grooteplein 10, 6500 Nijmegen, HB Netherlands; 2 Department of Craniofacial Orthodontics, Chang Gung Memorial Hospital, Taoyuan branch, No. 123, Dinghu Road, Guishan District, Taoyuan, 333 Taiwan; 3 Radboudumc 3D Lab, Radboud University Medical Center, Geert Grooteplein 10, 6500 Nijmegen, HB Netherlands

**Keywords:** Cleft, Congenital deformities, Dentofacial deformities, Orthognathic surgical procedures, Three-dimensional

## Abstract

**Objectives:**

To assess the surgical accuracy of 3D virtually planned orthognathic surgery among patients with and without cleft.

**Materials and methods:**

This retrospective cohort study included cleft and non-cleft patients with class III malocclusion who underwent bimaxillary surgery. CBCT scans were acquired before and immediately after surgery. 3D virtual surgical planning (VSP) was performed using CBCT and digitalized dentition data. All orthognathic surgeries were performed by the same surgeons using interocclusal splints. The primary outcome variable was surgical accuracy, defined as the difference between the planned and surgically achieved maxillary movements, quantified in six degrees of freedom. Analysis of covariance was used to test for intergroup differences in surgical accuracy after correcting for differences in the magnitude of planned surgical maxillary movements.

**Results:**

Twenty-eight cleft and 33 non-cleft patients were enrolled, with mean ages of 18.5 and 25.4 years, respectively (*P*=0.01). No significant gender difference was present between the groups (*P*=0.10). After adjustment for small differences in surgical movements, no significant differences in surgical accuracy were observed between cleft and non-cleft patients.

**Conclusion:**

The present study demonstrates that high surgical accuracy in maxillary movements can be achieved in both cleft and non-cleft patients using VSP and interocclusal splints.

**Clinical relevance:**

Orthognathic cases with cleft can be performed with 3D VSP to obtain a satisfactory surgical accuracy.

## Introduction

Cleft of the lip and palate are congenital dentofacial deformities. The treatment of cleft lip and palate deformities can be extensive and challenging for the surgeon due to anatomic and technical complexities that arise from multiple surgeries. As part of the total cleft treatment, most of the cleft patients require orthognathic surgery to correct maxillary hypoplasia and to improve oral functions and facial esthetics [1, 2]. Orthognathic surgery with cleft patients presents a greater clinical challenge compared to non-cleft patients because of the evident maxillary hypoplasia in all dimensions, as well as soft tissue scarring from previous surgeries such as lip correction, closure of the palate, and pharyngoplasty [3].

In recent years, three-dimensional (3D) virtual surgical planning (VSP) is becoming the clinical standard in orthognathic surgery. Multiple studies have used voxel-based matching to superimpose the preoperative and postoperative cone beam computed tomography (CBCT) scans for assessing surgical accuracy and for identifying factors that may influence or affect the feasibility of 3D VSP [4, 5]. Recent studies reported that the accuracy of maxillary positioning in non-cleft patients using a computer-aided design/computer-aided manufacturing (CAD/CAM) splint derived from 3D VSP is within the range of 1 to 2 mm [6, 7]. Studies with similar study design involving cleft patients demonstrated that the surgical accuracy of maxillary positioning varied between 1.48 and 2.75 mm [8, 9]. Anterior–posterior (A/P) translations remained challenging to achieve during surgery in cleft patients [8]. However, there is limited data in the existing literature regarding the achievability of 3D virtually planned maxillary movements, particularly in the context of a direct comparison between cleft and non-cleft patients.

The purpose of this study was to compare the surgical accuracy of maxillary positioning in cleft and non-cleft patients who underwent bimaxillary surgery, using CAD/CAM interocclusal splint between cleft and non-cleft patients. Furthermore, the study aimed to assess the influence of cleft type, posterior impaction, and history of a pharyngoplasty on the surgical accuracy in the cleft group.

## Material and methods

This retrospective cohort study included cleft and non-cleft patients with a class III malocclusion who underwent bimaxillary osteotomy between 2017 and 2022 at the Department of Oral and Maxillofacial Surgery in Radboud University Medical Center (Nijmegen, the Netherlands). Patients were enrolled consecutively. The inclusion criteria were non-syndromic patients with a dysgnathia requiring bimaxillary osteotomy that consisted out of bilateral sagittal split osteotomies (BSSO) and a one-piece Le Fort I osteotomy. Preoperative orthodontic treatment, closed cleft lip and palate (when applicable), a minimum of 22 teeth, and the use of 3D VSP derived CAD/CAM splints were also mandatory. The exclusion criteria were previous orthognathic surgery, except for surgically assisted rapid maxillary expansion (SARME), suboptimal condyle seating on the pre- and/or postoperative CBCT scans, and/or a history of facial trauma.

This study was performed in accordance with the protocol of the World Medical Association Declaration of Helsinki on medical research ethics and was approved by the Institutional Review Board (CMO Arnhem-Nijmegen, #2022-16078). All data were pseudonymized prior to analysis.

### Data collection

The CBCT scans were acquired using a standard extended height CBCT scanning protocol (FOV 23 × 17 cm at 120 kV and 0.4-mm isotropic voxel size) with a KaVo 3D Exam CBCT scanner (KaVo, Biberach, Germany). Patients were scanned both 4 weeks prior to the surgery and within 1 week postoperatively. During the scanning, patients were asked to maintain in natural head position while seated, with their facial muscles relaxed and eyes open. Following surgery, patients were scanned in centric occlusion, while the surgical splint was still in place. Subsequently, the CBCT data were exported in DICOM format.

### 3D planning and surgical procedure

3D VSP was performed in all patients using IPS CaseDesigner (KLS Martin, Tuttlingen, Germany). The CBCT data were used as image data for soft and bone tissue. Detailed dentition data were imported as STL files from digitalized plaster casts or intra-oral scans (IOS) (TRIOS® 3, 3Shape™, Copenhagen, Denmark).

A 3D-augmented head model was generated by fusing the CBCT scans and dentition data. A virtual bimaxillary osteotomy was performed on this model with the final occlusion being determined using the virtual occlusion tool within the IPS CaseDesigner software. The maxilla and mandible were repositioned to obtain a harmonious soft tissue facial profile, which was simulated in real-time through the software. Intermediate and final interocclusal splints were designed and fabricated based on the 3D VSP to transfer the surgical plan to the patient during the operation.

3D VSP and surgeries were performed by an experienced surgeon or under the direct supervision. The maxilla was operated on first in all cases. Following Le Fort I osteotomies, a manual down fracture and mobilization of the maxilla by using Rowe forceps, the intermediate interocclusal wafer, and a nasion pin were used to reposition the maxilla. The maxilla was then fixated with titanium miniplates (Stryker 1.7 midface system, Freiburg, Germany). Subsequently, bilateral sagittal split osteotomies (BSSO) were performed according to the Hunsuck modification [10]. The final interocclusal wafer was used to reposition the mandible as planned. For fixation purposes, miniplates and monocortical screws were utilized (Stryker 2.0 MP system, Freiburg, Germany). The interocclusal splint and tight elastics were removed during the first postoperative follow-up, scheduled 1 week after surgery. Subsequently, guiding elastics were employed, and postoperative orthodontic treatment was initiated.

### Analysis of study outcomes

The primary outcome variable was the surgical accuracy of the maxillary repositioning in six degrees of freedom: sagittal, vertical, and transverse translations (mm) in combination with pitch, roll, and yaw rotations (°). The surgical accuracy was defined as the 3D spatial difference of the maxilla between the 3D VSP and postoperative CBCT. The primary predictor variable was the presence of cleft. The secondary predictor variables were planned posterior maxillary impaction, history of pharyngoplasty, and type of cleft (unilateral or bilateral). Planned posterior impaction was defined as any cranial movement of both first maxillary molars in the 3D VSP. Covariates such as gender, age, and the magnitude of the planned movements were included. 3D analysis of the surgical accuracy was performed by one observer (DB) using the OrthoGnathicAnalyser (OGA 2.0) according to the following steps [11, 12]:

1. The upper incisor point, defined as the most mesial point on the incisal edge of the right upper central incisor (11), served as a reference point for calculating the translations and rotations.
2. The preoperative 3D virtual head model was orientated in the natural head position that was previously used in IPS Casedesigner software.
3. The pre- and postoperative 3D virtual head models were aligned via voxel-based matching upon the anterior cranial base.
4. The .STL files of VSP were used to translate the preoperative virtually osteotomized maxilla to the 3D planned position in IPS CaseDesigner using surface-based matching. The resulting transformation matrix, containing the maxillary translations and rotations, was saved.
5. The maxilla from the pre- and postoperative CBCT data was translated from the preoperative position to the postoperative position using voxel-based matching. Then again, the resulting transformation matrix was saved.
6. The final step was to calculate the surgical accuracy. To do so, the transformation matrices from previous steps were converted in six degrees of freedom.

### Statistics

Statistical analysis was performed in SPSS (version 29, IBM Corp., Armonk, NY, USA). Demographic characteristics of the study population and each subgroup were explored using descriptive statistics. Kolmogorov–Smirnov test was used to test the normal distribution of the primary outcome variable. Absolute mean errors and intra-class correlations (ICC) were calculated to assess the measurement errors and intra-rater reliability. ANCOVA with post hoc Bonferroni correction was performed to test for intergroup differences between the cleft and non-cleft group while accounting for covariates. Linear regression was used to test for statistical significant differences between subgroups. The dependent variable was the surgical accuracy of the front–back movement, and factors incorporated as independent variables were cleft condition, type of cleft, pharyngoplasty, and posterior maxillary impaction.

## Results

Sixty-one patients are enrolled in this study, comprised of 34 males and 27 females, and a mean age of 22.3±7.1(SD) years. The cleft group comprised of 28 patients, 15 males, and 13 females, with a mean age of 18.5±1.4 years. Of 28 patients in the cleft group, 21 patients (75%) underwent a pharyngoplasty, 7 patients (25%) had a bilateral cleft, and in 7 patients (25%), a surgical posterior maxillary impaction was performed. The non-cleft group comprised of 33 patients, 19 males, and 14 females, with a mean age of 25.4±8.4 years, and in 15 patients, posterior impaction was performed. No statistically significant difference in gender distribution was present between the cleft and non-cleft group (*p*=0.75). The age difference between the cleft and non-cleft group was statistically significant (6.9 years, *p*<0,001).

Concerning the measurement error and ICC, the maximum measurement error (0.03 ± 0.96°) was observed for the pitch of the maxilla. The lowest ICC observed was 0.944 and concerned the roll of the maxilla. Both indicated a low measurement error and a high intra-rater reliability.

**Fig. 1:**
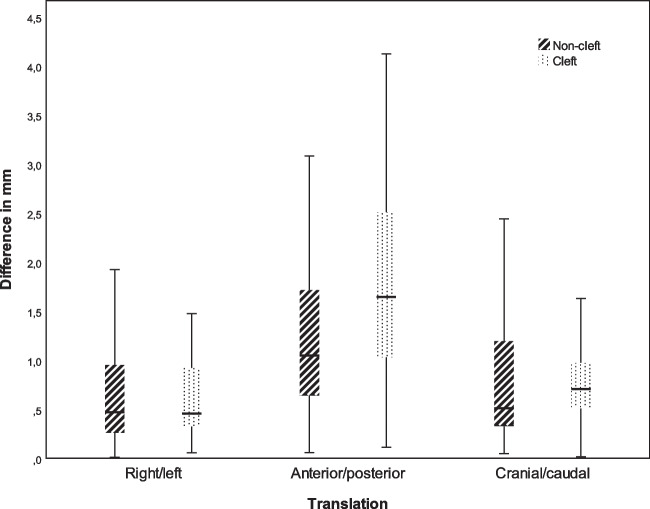
Boxplot^a^ of the absolute differences between the planned and achieved translational movements of the maxilla in the non-cleft and cleft group. ^a^The lower(Q1) and upper (Q4) quartiles are represented by the whiskers

**Fig. 2:**
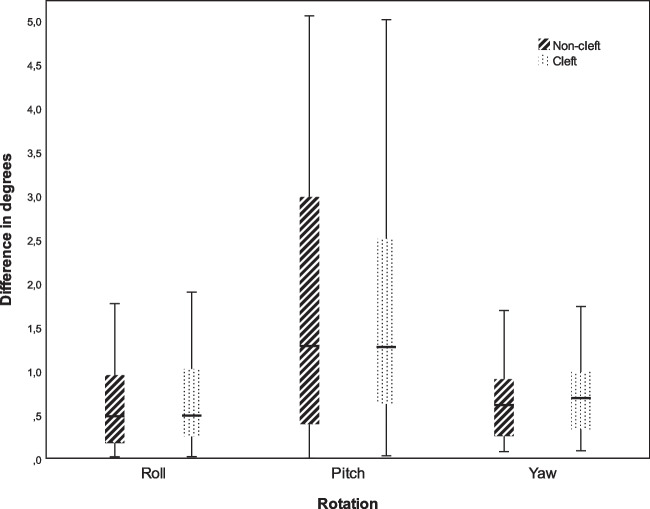
Boxplot^a^ of the absolute differences between the planned and achieved rotational movements of the maxilla in the non-cleft and cleft group. ^a^The lower(Q1) and upper (Q4) quartiles are represented by the whiskers

The surgical accuracy in the cleft and non-cleft group in six degrees of freedom are displayed in Table 1 (Figs. 1 and 2). No statistically significant differences were found between the groups. The planned movements of the maxilla in six degrees of freedom are displayed in Table 2 for both the cleft and non-cleft group. A statistically significant difference was found for the planned yaw between the cleft (−1.97±3.20°) and non-cleft patients (−0.16±1.91°; *p*=0.01). No further statistically significant differences in virtually planned maxillary movements were found. Linear regression showed a poor fit (*R*^2^=0.11) and no statistically significant influence of factors (cleft, type of cleft, pharyngoplasty and posterior impaction) on the surgical accuracy of the front–back movement.

**Table 1:** Comparison of the absolute differences (surgical accuracy) between the planned and achieved maxillary movements of the cleft and non-cleft group

		Non-cleft (*n* = 28)	Cleft (*n* = 33)	*P*-value
		Mean ± SD	Mean ± SD	
Translations (mm)	Left/right	0.69±0.54	0.69±0.66	0.96
	Anterior/posterior	1.92±1.19	1.38±1.07	0.07
	Cranial/caudal	1.20±1.20	0.82±0.71	0.13
Rotations (^0^)	Pitch	0.69±0.56	0.81±0.82	0.53
	Roll	1.85±1.71	1.74±1.59	0.80
	Yaw	0.99±0.93	0.88±0.83	0.61

*Statistical significance (*P* > 0.05)

**Table 2:** Comparison of the planned movements of the cleft and non-cleft group

		Non-cleft (*n* = 28)	Cleft (*n* = 33)	*P*-value
		Mean ± SD	Mean ± SD	
Translations (mm)	Left/right	−0.63±1.86	−0.09±1.01	0.16
	Anterior/posterior	−5.87±1.70	−5.16±1.34	0.08
	Cranial/caudal	−1.61±1.75	−1.22±1.84	0.39
Rotations (^0^)	Pitch	0.69±2.16	0.37±1.40	0.49
	Roll	2.71±2.56	2.34±2.87	0.61
	Yaw	−1.97±3.20	−0.16±1.91	0.01*

*Statistical significance (*P* > 0.05)

## Discussion

The aim of this study was to assess the surgical accuracy of 3D virtually planned orthognathic surgery among patients with and without cleft. The results of the present study showed no statistically significant difference in the six degrees of freedom of surgical accuracy between the cleft and non-cleft group. All translational degrees of freedom differed less than 2 mm, and all rotational degrees of freedom differed less than 4 degrees which is considered clinically acceptable [13]. The overall high surgical accuracy achieved in the study population was likely a consequence of the more than 10 years of experience in planning orthognathic surgeries with soft tissue–based 3D VSP, adequate mobilization of the maxilla, and sufficient removal of osseous interferences in posterior maxilla during surgery and the use of voxel-based quantification to measure surgical accuracy.

The results of the present study are in line with the current literature concerning the surgical accuracy of orthognathic surgery in patient with cleft and without cleft. To the best of the authors’ knowledge, only one other study has directly compared surgical accuracy in occlusal splint-based orthognathic surgery between cleft and class III non-cleft patients. Bollato et al. quantified surgical movements in terms of changes in SNA and SNB angles, without stating whether the absolute planned surgical movements in both groups [3]. Despite significant differences in methodology, the conclusion of the present study was in line with these previous studies, demonstrating a comparable surgical accuracy between cleft and non-cleft class III patients.

The magnitude of the surgical accuracy in six degrees of freedom of the present study concerning both the cleft and class III non-cleft group is in line with current literature or studies that have either investigated the surgical accuracy in cleft patients or in non-cleft patients [8, 9, 14]. All studies found that the largest surgical inaccuracy was present in the anterior/posterior, cranial/caudal translations, or pitch rotations. This similar trend was observed in the present study.

The surgical achievability of anterior/posterior translation has always been one of the least accurate surgical movement in orthognathic surgery among cleft and non-cleft patients [8, 15, 16]. Many studies reported that a larger maxillary advancement is correlated with more surgical inaccuracy. As the planned maxillary advancement of both groups in the present study had a mean magnitude of more than 5 mm, which is larger than most previous studies, more surgical inaccuracy in this direction could be expected. Although no statistically significant difference was found in maxillary advancement between cleft and non-cleft patients, our surgeons experienced more stiffness of soft tissue surrounding the maxilla during mobilization with cleft patients compared to non-cleft patients, especially in the posterior maxilla. A history of pharyngoplasty was hypothesized to be a factor that may impede a proper maxillary advancement due to an increased soft tissue resistance in the posterior maxilla as result of an altered anatomy and presence of more scar tissue. However, the subgroup analysis showed no significant influence of pharyngoplasty on the surgical achievability of maxillary advancement.

An explanation for the discrepancy in the cranial/caudal plane could be that the interocclusal splint only accounts for the transfer of five degrees of freedom, excluding the vertical plane, as this is determined intra-operatively by measuring the distance between the nasion pin and the incisal point of the upper right central incisor. The surgical accuracy of the vertical jaw displacement is, therefore, more prone to operator error. This could be one of the explanations for the inaccuracy that is seen in the vertical plane. In addition, patients with cleft lip and palate often have scarring of the upper lip which is more difficult to simulate in 3D VSP [17]. This could, in turn, lead to a suboptimal 3D VSP concerning the planned vertical translations, affecting the surgical accuracy in the vertical plane. Furthermore, the dental show is used intra-operatively to determine the amount of final vertical displacement of the maxilla. As this is the leading parameter, it could differ from the planned vertical maxillary movement in 3D VSP.

Challenges in achieving a high surgical accuracy regarding the planned clockwise pitch are often described in literature, as bony interferences between the posterior maxilla and pterygoid region impede a proper posterior maxillary vertical impaction [18, 19]. Extensive surgical experience and meticulous execution of the removal of bony interferences is necessary to reduce this surgical inaccuracy [6]. The findings of the present study demonstrated that a cleft condition, i.e., scarring in the palatal region, and as result of pharyngoplasty, did not surgically complicate surgical posterior maxillary impaction. Removing posterior bony interferences is still the key in obtaining the planned maxillary impaction.

The planned yaw difference between the cleft and non-cleft groups is a finding that is worth to note. This may be attributed to the common absence of a lateral incisor in case of unilateral cleft lip and palate. Orthodontically, there are two strategies to manage this in presurgical orthodontics. The first option involves orthodontic closure of the edentulous space to preclude the need for a prosthetic replacement of the absent lateral incisor. However, unilateral space closure might result in arch length discrepancy, asymmetric maxillary dental arch, and a consequential upper dental midline shift [20]. The second option is space opening for a prosthetic replacement of the missing lateral incisors, which results in a more symmetric dental arch. As there is often a preference in our orthodontic team for closing the gap orthodontically, there was often a maxillary arch form discrepancy between the left and right side prior to surgery. In order to coordinate the mandibular arch to the asymmetric maxillary arch, a subsequent yaw movement of the mandible is required. This yaw movement of the mandible can lead to interference between the proximal and distal segments and can potentially lead to posterior asymmetry in the mandibular angle region. Thus, the bimaxillary complex is often adjusted with a counteracting yaw movement to reduce the undesired mandibular movement. This adjustment accounts for the statistically significant difference in planned yaw between the cleft and non-cleft groups. There was no statistically significant difference in the achieved yaw movement between the groups, which demonstrated the surgical ability of achieving the desired yaw movement. This finding underlines the importance of 3D VSP in orthognathic surgery, particularly for the cleft group. Despite arch length discrepancies and the potential for esthetically unpleasing outcomes, 3D VSP facilitates achieving satisfactory soft tissue–based results.

A strength of this study is twofold: (1) the use of a three-dimensional semi-automated voxel-based approach to quantify maxillary movement eliminated the necessity of identifying corresponding cephalometric landmarks on different scans, thus providing results with a high accuracy and reproducibility[11]; and (2) the presence of comparable demographic characteristics and surgical movements between the cleft and non-cleft group, reducing the heterogenicity in intergroup comparison to a minimum. In this study, we included both unilateral and bilateral cleft lip and palate patients in the cleft group, but patients with unilateral cleft lip and palate tend to have more planned yaw correction and would warrant further study.

A limitation of this study was the limited power for the primary outcome variable, primarily due to the modest size of the study population. This can result in not detecting small differences (less than 0.5 mm or degrees) in surgical accuracy between the cleft and non-cleft groups. However, it is important to note that previous studies have shown that differences greater than 2 mm or degrees are considered clinically relevant by patients and surgeons [21]. From this perspective, a small difference in surgical accuracy, i.e., 0.5 mm in the anterior–posterior direction, would not be considered clinically relevant, even if statistically significant. In addition, the small study population in combination with the presence of outliers in surgical accuracy (particularly in the anterior–posterior direction) may limit the generalizability of the results of the present study.

In conclusion, the present study showed that a comparable high surgical accuracy can be achieved in both cleft and non-cleft patients, by using 3D VSP and interocclusal splints. Management of cleft patients requires careful consideration during the orthodontic phase, 3D VSP, and surgical process. 3D VSP can aid the surgeon to overcome challenges associated with cleft orthognathic surgery and aid the surgeon to achieve comparable surgical jaw movements as in non-cleft patients.
